# Durability Performance of Basalt Fiber-Reinforced Concrete Subjected to Sulfate–Magnesium Combined Attack

**DOI:** 10.3390/ma17051128

**Published:** 2024-02-29

**Authors:** Henghui Fan, Cheng Wang, Yiqi Hu, Gaowen Zhao

**Affiliations:** 1 College of Water Conservancy and Architectural Engineering, Northwest A&F University, Xianyang 712100, China; yt07@nwsuaf.edu.cn (H.F.); wchgghwzy@163.com (C.W.); 2 School of Highway, Chang’an University, Xi’an 710064, China; 3 Key Laboratory for Special Area Highway Engineering, Ministry of Education, Chang’an University, Xi’an 716400, China

**Keywords:** cast-in situ concrete, degradation mechanism, sulfate attack, magnesium attack, basalt fiber

## Abstract

In salt lake areas, cast-in situ concrete structures are subjected to long-term corrosion by sulfate and magnesium ions. The properties of concrete can be improved by adding materials like basalt fiber (BF). To investigate the degradation process and mechanism of cast-in situ concrete with premixed BF under the dual corrosion of sulfate and magnesium salts, concrete with a content of BF ranging from 0 to 0.5% was prepared. Specimens were subjected to different internal and external corrosion conditions and immersed for 180 days. Dimension, mass, and appearance changes at different immersion times were recorded. The compressive and flexural strength of the specimens were tested and continually observed throughout the immersion time. Mineral and microstructural changes at different immersion times were determined by the XRD, TG, and SEM analysis methods. Results indicated that external sulfate–internal magnesium combined attack had a significant negative effect on the early strength. The compressive and flexural strength of the corroded specimens decreased by 17.2% and 14.1%, respectively, compared to the control group at 28 days. The premixed magnesium ions caused the decomposition of the C-S-H gel, resulting in severe spalling and lower mechanical properties after immersing for a long time. As the BF can inhibit crack development, the properties of the concrete premixed with BF were improved. Specimens exhibited superior performance at a BF content of 0.5%, resulting in a 16.2% increase in flexural strength. This paper serves as a valuable reference for the application of basalt fiber-reinforced concrete under the challenging conditions of sulfate–magnesium combined attack.

## 1. Introduction

Sulfate attack poses a notable risk to concrete structures in salt-lake and saline areas [1,2,3]. Previous studies have classified sulfate attacks on concrete into: (1) Chemical corrosion: The invading sulfate ions undergo chemical reactions with cement hydration products, generating corrosion products including gypsum and ettringite. The accumulation of corrosion products causes expansion and cracks inside the concrete matrix, and the development of cracks further increases the erosion channels of sulfate, eventually causing the deterioration of concrete structures [4]. (2) Crystallization corrosion: when the sulfate solution in the capillary pores of concrete is oversaturated, there will be crystallization precipitations and crystallization pressure that induce damage to the concrete structures [5,6]. Regarding the mechanism of crystallization corrosion, Scherer considered that the main reason for the damage is the expansion of the solid phase, attributed to the variation of crystal-bound water content in crystalline salt [7]. Flatt thought the growth process of crystals would generate a considerable crystallization pressure in the pores, resulting in concrete deterioration [8]. (3) Chemical-crystallization corrosion: Concrete is subjected to chemical and crystallization stresses, causing more severe degradation [9].

Mg^2+^ is widely distributed in groundwater and soil in salt-lake and offshore areas [10]. Currently, it is believed that Mg^2+^ can expedite the degradation of concrete induced by SO_4_^2−^, causing more severe cracks and spalling [11]. The chemical reaction of Mg^2+^ and calcium hydroxide (CH) would lower the pH of the pore solution, making the C-S-H gel unstable, and the leaching of Ca^2+^ leads to the generation of M-S-H with low strength, significantly impacting the formation of the gel structure [12,13]. Liu et al. found that the structure of M-S-H is contingent on the initial Ca/Si ratio of C-S-H, where C-S-H gel with a lower Ca/Si ratio is more susceptible to the corrosion caused by Mg^2+^ [14]. As for external magnesium sulfate corrosion, Mg^2+^ has the capability to react with the hydration products of cement to form magnesium hydroxide (Mg(OH)_2_), which would further decompose to generate gypsum [15]. In addition, magnesium sulfate corrosion can also result in the decomposition of the C-S-H gel, promoting the generation of gypsum and ettringite [16,17]. However, some studies have found that magnesium in the environment can delay the degradation induced by sulfate [18,19]. Li et al. found that magnesium decreases the contact area of cement with water, slowing the hydration rate and diminishing the production of hydration products [20]. Under the corrosion of magnesium sulfate, a protective layer would be formed on the surface of concrete [21]. The structures of magnesium-induced corrosion products are mainly flaky and layered, which are embedded in the matrix rather than accumulated in the pores, therefore blocking the diffusion paths for harmful ions.

Previous research has focused extensively on the mechanical properties and deterioration mechanism of precast concrete (standard curing for 28 days) subjected to sulfate attack. Nevertheless, cast-in situ concrete structures find widespread application in bridges and roads in salt-lake and coastal areas [22]. In these areas, cast-in situ concrete comes into contact with corrosive ions immediately after being put into the environment, exerting a substantial impact on the strength and durability of the concrete. Currently, the demand for concrete is staggering [23,24]. Due to the scarcity of fresh water in some areas and the limitations of river sand extraction, the use of groundwater and sea sand to prepare concrete is being explored [25,26,27]. Groundwater in coastal and inland areas contains considerable content of SO_4_^2−^, Cl^−^, Mg^2+^, and other corrosive ions [28], and the surface or pores of sea sand always contain salt crystals, which dissolve during the mixing process. Therefore, using groundwater or sea sand to prepare concrete would cause internal corrosion. The scarcity of fresh water in salt lake areas necessitates the use of lake water or groundwater for concrete mixing, leading to internal corrosion. Meanwhile, the high content of sulfate ions in the environment results in severe degradation. The combined external–internal attack poses a significant challenge to concrete structures. However, there is limited research on the deterioration mechanism of cast-in situ concrete subjected to the composite attack at present. Thus, it is necessary to conduct research in this area.

Research found that incorporating fibers into concrete can enhance its mechanical properties and durability, particularly in challenging environments [29]. The influence of fibers on concrete includes inhibiting the development of cracks, reducing the detrimental effect of inherent defects on mechanical properties, and withstanding stress and deformation, thereby enhancing the toughness of the concrete [30,31,32]. Basalt fiber (BF) is an inorganic fiber material with high ultimate elongation, high elastic modulus, excellent tensile strength, and strong chemical stability [33]. The density of BF is close to concrete, and evenly distributed BF can be closely connected with cement slurry and aggregates. With excellent toughness, BF inhibits the formation of micro-cracks in the concrete and improves its compactness [34]. Many studies indicate that the use of BF enhances concrete’s resistance to sulfate attack [33,35,36,37,38,39]. Wang et al. [38] found that an appropriate amount of BF can limit the expansion of pores and cracks, thereby enhancing corrosion resistance, but excessive BF would adversely affect durability. Wu et al. [39] observed that the decrease in strength and mass loss of basalt fiber-reinforced concrete exposed to sulfate attack is smaller than that of ordinary concrete. At present, there is no definitive conclusion on whether BF can enhance the performance of concrete under external–internal combined attack. Thus, performing a study in this area would be highly meaningful.

The aims of this study are to investigate the deterioration mechanism of cast-in situ concrete exposed to external sulfate–internal magnesium combined attack, and to research the enhancing effect of different contents of BF on the specimen properties. Currently, internal corrosion of concrete mainly considers pyrite and gypsum [13,40], and there is nearly no research conducted on the internal corrosion caused by magnesium salts. Additionally, the deterioration mechanisms of cast-in situ concrete are different from those of precast concrete. In this study, 3% magnesium sulfate was premixed into the water to simulate internal corrosion, and concrete with 0–0.5% content of BF was prepared. Subsequently, all the specimens were placed in distilled water and 10% sodium sulfate solution, respectively. To reveal the degradation process, the change in appearance, mass, dimension, and mechanical properties of each specimen were measured and compared at various immersion times. Powder from different depths was obtained by drilling, and the sulfate concentration at each depth was determined through chemical titration. To analyze the mineral change and microstructure of the specimen, scanning electron microscopy (SEM) with energy dispersive spectroscopy (EDS), X-ray diffraction (XRD), and thermogravimetry-derivative thermogravimetry (TG/DTG) were used in the present study. Improving the durability of cast-in situ concrete in salt lake areas saves a lot of costs and reduces waste emissions, which is of great significance to ecology and the economy. This study serves as a valuable reference for the application of basalt fiber-reinforced concrete under challenging conditions.

## 2. Materials and Methods

### 2.1. Materials

Jidong Portland cement (P.O. 42.5) was employed in the current experiment to prepare concrete specimens. The basic chemical composition of the cement was tested by XRF, and the results are presented in Table 1. Xingping river sand with a fineness modulus of 2.6 was selected, as fine aggregates and pebbles from Jingyang quarry with a particle size of 5–10 mm were used for coarse aggregates in this study. AR Na_2_SO_4_ and MgSO_4_ produced by the Damao Chemical Reagent Factory were used to prepare corrosive solutions and specimens. The 12 mm length of short-cut BF produced by Shanghai Chenqi Chemical Technology Company was used to mix in the concrete, and the physical properties are also shown in Table 1. 

### 2.2. Testing Methods

#### 2.2.1. Specimen and Solution Preparation

In the specimen preparation process, the water–cement ratio is 0.46, and the mixture proportion of water, cement, sand, and pebbles is 220:478:629:983. To distribute the BF evenly in the concrete, fine aggregate and coarse aggregate were weighed and dry-mixed for 30 s at first. Then, the weighed BF was put into the mixer evenly in batches and mixed for 60 s. Finally, the cement and water were mixed for 120 s. To simulate the internal corrosion problem induced by magnesium salt in salt-lake or saline areas, 3% MgSO_4_ (by mass ratio) was added to distilled water before being put into the mixer. Na_2_SO_4_ was used for the preparation of sulfate solution with a content of 10% in distilled water. BF can enhance the performance of the specimens, but it also significantly affects workability. Therefore, the premixed 0.1%, 0.3%, and 0.5% (by volume ratio) content of BF in the present study was determined by the published papers [35,41]. Freshly mixed concrete was cast in 40 mm × 40 mm × 160 mm and 100 mm × 100 mm × 100 mm molds and adequately vibrated on a vibration table. All specimens were cured for 2 h at 20 °C and 95% relative humidity, then put into different solutions with molds, and demolded after 24 h. It is crucial to emphasize that the initial hardening of the surface of the concrete specimen requires a curing time of 2 h to avoid the disintegration of fresh paste in aggressive solutions. Five types of specimens were prepared, namely the control group (hereinafter referred to as C), samples premixed with 3% MgSO_4_ (hereinafter referred to as M), samples premixed with 3% MgSO_4_ and 0.1% BF (hereinafter referred as 1F), samples premixed with 3% MgSO_4_ and 0.3% BF (hereinafter referred as 3F), and samples premixed with 3% MgSO_4_ and 0.5% BF (hereinafter referred as 5F). The corresponding immersion solutions were added before the names of the specimens, where D represents distilled water and S represents 10% sulfate solution. Details of the different specimens and solutions are provided in Table 2 and all specimens were immersed below the liquid level.

#### 2.2.2. Dimension and Mass Change

The average dimension of specimens was determined three times at different immersion times by dial indicator and comparator (produced by Hitachi, Japan) with a copper limiter embedded in 40 mm × 40 mm × 160 mm specimens. The measurement was in accordance with Chinese standard JC/T 603-2004, with an accuracy of 0.001 mm. Mass changes in specimens were recorded by weighing each specimen three times using an electronic scale with an accuracy of 0.001 g (produced by Meilen, Shenzhen, China). Dimension and mass changing ratios were calculated by (a_t_ − a_0_)/a_0_ (a_t_ is the current value and a_0_ is the initial value of size or weight).

#### 2.2.3. Compressive and Flexural Strength

The test was conducted according to EN 196-1:2005, and the compressive strength was determined by a TYE-2000H (produced by China) compression-testing machine. The loading rate was settled at 0.5 MPa/s and the size of the test specimen was 100 mm × 100 mm × 100 mm. The flexural strength was determined by a TYE-6A (produced by China) flexure-testing machine. The loading rate was settled at 0.05 MPa/s, and the size of the test specimen was 40 mm × 40 mm × 160 mm. To obtain an accurate result, three parallel specimens were used to obtain an average strength value. Hence, a total of 420 samples were tested, with 210 samples evaluated for compressive strength and 210 samples for flexural strength.

#### 2.2.4. Microstructural and Mineral Analysis

Microstructural images of different specimens at different immersion times were obtained by SEM using the Hitachi S-4800 system (produced by Japan). EDS analysis was also conducted to further analyze the specific composition of observed crystals during SEM observation. A golden coating was covered on the specimens. The mineral composition of different specimens at different corrosion times was determined by XRD using an AXS D8 Advance X-ray diffractometer (produced by Germany). TG analysis was performed using a TA Discovery SDT 650 system (produced by the USA) to further confirm the corrosion products during immersion time. Samples for the SEM test were obtained from drilled core samples located 5 mm beneath the exposed surface of the concrete specimens. Powder samples for TG and XRD analysis were obtained by drilling 5 mm beneath the exposed surface. The samples were passed a 0.36 mm sieve and then dried at 40 °C to a constant weight [42].

#### 2.2.5. Sulfate Concentration

**Figure 1:**
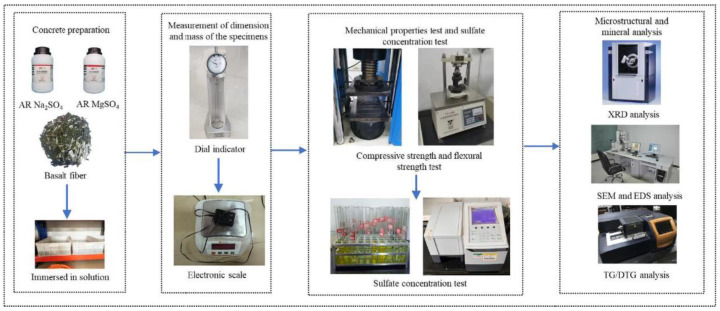
The flow chart of the experiment.

The sulfate concentration was titrated with powder samples from a 100 mm × 100 mm × 100 mm specimen. Sulfate concentrations at various depths beneath the exposed surface of specimens were determined by chemical titration at the corresponding age. Powder was drilled at intervals of 5 mm perpendicular to the exposed surface, resulting in a total of 5 sets of samples. Obtained powder samples were dried in an oven at 60 °C until the weight of the specimen was constant. Then, the powder samples were weighed and mixed with 50 mL of distilled water for 48 h; subsequently, the sulfate content was determined by chemical titration [43]. In summary, the process of the experiment is shown in Figure 1.

## 3. Results

### 3.1. Appearance Change

**Figure 2:**
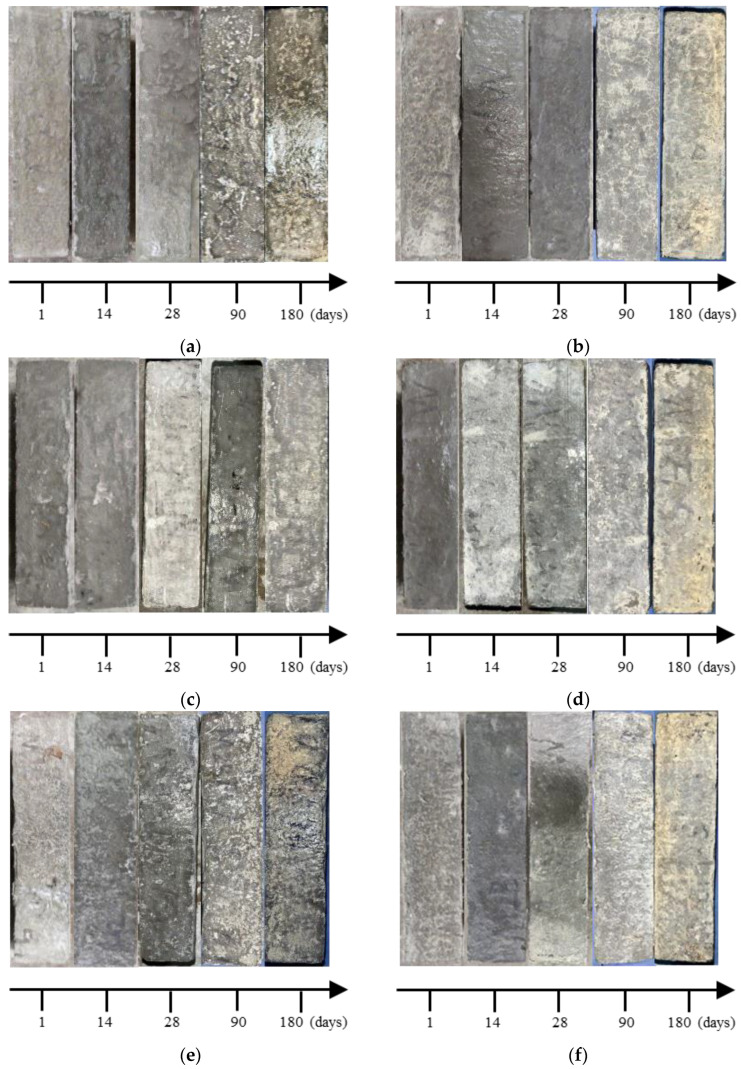

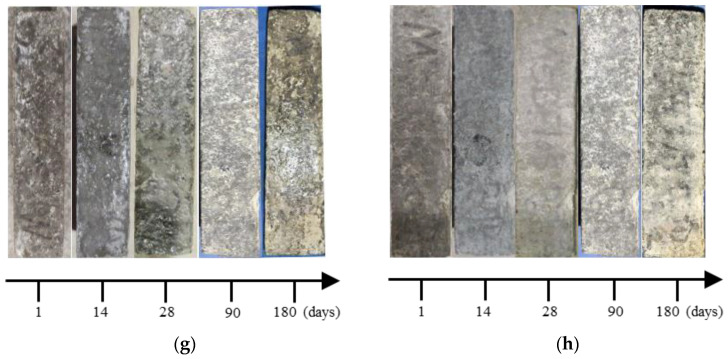
The appearance changes of specimens under different immersion times: (**a**) D-M, (**b**) S-M, (**c**) D-1F, (**d**) S-1F, (**e**) D-3F, (**f**) S-3F, (**g**) D-5F, and (**h**) S-5F.

Images of specimens premixed with corrosive salts at different times are shown in Figure 2. The degradation extent of cast-in situ concrete is different under these two corrosion conditions. As the immersion time increases, the surfaces of D-M and S-M become sanded due to the premixed corrosive ions. For specimens immersed in distilled water, the surfaces remain relatively intact at 28 days. However, after 90 days, peeling and falling off of the cement slurry are observed at the surface of the specimens, while for specimens immersed in sulfate solution, the surfaces are intact at the early stage as well. At 90 days, the surfaces of the specimens exhibit whitening, which is attributed to the salt crystallization. The degradation of concrete induced by sulfate solution is more pronounced, the surfaces become coarser, and more powder falls off with prolonged immersion time. After 90 days, the corners of the specimens are damaged at first, while the central parts are still intact. At 180 days, more cracks develop at the corner of the specimens, surface spalling becomes more severe, and denser holes occur. Premixed salts generate corrosive products in the early stage of the hydration process, so specimens are more severely damaged by the external sulfate–internal magnesium combined attack. From Figure 2, it is clear that specimens with premixed BF suffer less deterioration, the degree of spalling is reduced, and the integrity of corners is improved.

### 3.2. Microstructure and Mineral Changes

Different corrosion conditions can markedly influence the degradation and mechanical properties of concrete structures. The reason can be attributed to the change in microstructures and mineral composition. Thus, it is crucial to make clear the microstructures and mineral changes during the immersion time.

#### 3.2.1. Mineral Changes

**Figure 3:**
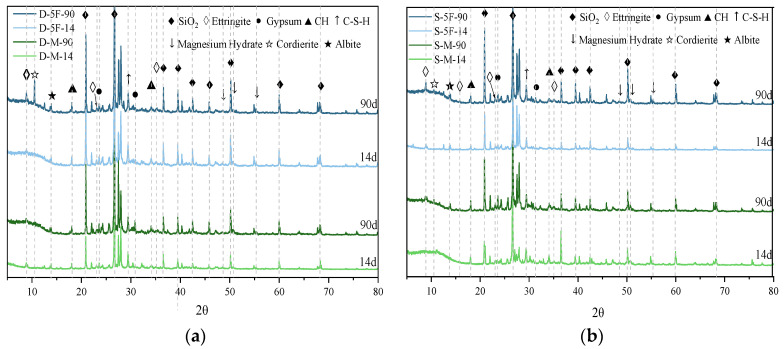
XRD results of specimens immersed in (**a**) distilled water, and (**b**) 10% sulfate solution for different immersion times. The numerals represent the immersion times.

**Figure 4:**
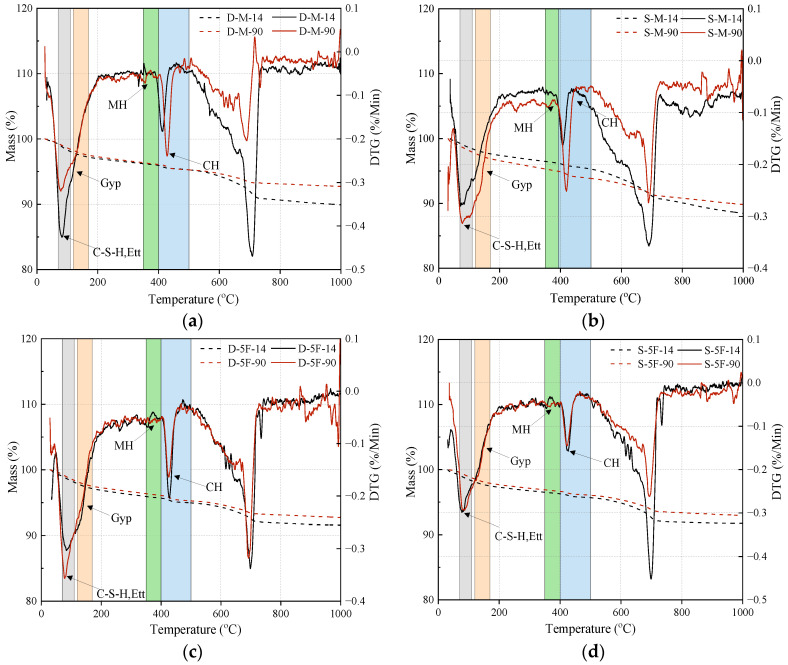
Results of TG/DTG analysis of each specimen under different immersion times: (**a**) D-M, (**b**) S-M, (**c**) D-5F, and (**d**) S-5F. Ett represents ettringite, Gyp represents gypsum, and MH represents magnesium hydrate.

Mineral changes in specimens immersed in different corrosive conditions were determined by XRD and TG tests, respectively. D-M, S-M, D-5F, and S-5F were selected as the representatives of external sulfate–internal magnesium combined attack and the influence of BF on cast-in situ concrete. The results of XRD are illustrated in Figure 3 and TG results are provided in Figure 4.

It is important to note that the determination and identification of corrosion products rely on the reference PDF cards from Jade and information from published papers. From the XRD pattern in Figure 3, corrosion products including gypsum (Reference code: 21-0816), ettringite (Reference code: 41-1451), magnesium hydrate (Reference code: 82-0503), hydration products CH (Reference code: 76-0571) and C-S-H (Reference code: 33-0306), SiO_2_ (Reference code: 85-0798), and minerals from coarse aggregates including cordierite (Reference code: 13-0294) and albite (Reference code: 09-0456), can be detected. The diffraction peaks of gypsum and ettringite in specimens immersed in sulfate solution are higher than those immersed in distilled water. It is speculated that more corrosion products were generated under the external sulfate attack. Meanwhile, the reactions continue with the prolonged immersion time, and the peaks of corrosion products also become more pronounced. The corrosion reactions caused by sulfate are as follows [22]:

(1) $${\mathrm{Ca}(\mathrm{OH})}_{2}+{\mathrm{Na}}_{2}SO_{4}+2H_{2}O\to {\mathrm{CaSO}}_{4}\cdot {2H}_{2}O+2\mathrm{NaOH}$$

(2) $$3\mathrm{CaO}\cdot {\mathrm{Al}}_{2}O_{3}+3\left({\mathrm{CaSO}}_{4}\cdot 2H_{2}O\right)+26H_{2}O\to 3\mathrm{CaO}\cdot {\mathrm{Al}}_{2}O_{3}\cdot 3\mathrm{CaS}O_{4}\cdot 32H_{2}O$$

(3) $$3\mathrm{CaO}\cdot {\mathrm{Al}}_{2}O_{3}\cdot {\mathrm{CaSO}}_{4}\cdot 12H_{2}O+2\left({\mathrm{CaSO}}_{4}\cdot 2H_{2}O\right)+16H_{2}O\to 3\mathrm{CaO}\cdot {\mathrm{Al}}_{2}O_{3}\cdot 3\mathrm{CaS}O_{4}\cdot 32H_{2}O$$

(4) $$4\mathrm{CaO}\cdot {\mathrm{Al}}_{2}O_{3}\cdot 13H_{2}O+3\left({\mathrm{CaSO}}_{4}\cdot 2H_{2}O\right)+14H_{2}O\to 3\mathrm{CaO}\cdot {\mathrm{Al}}_{2}O_{3}\cdot 3\mathrm{CaS}O_{4}\cdot 32H_{2}O+\mathrm{CaO}\cdot H_{2}O$$

The premixed Mg^2+^ chemically reacts with the hydration products, resulting in the formation of the corresponding corrosion products. After the consumption of CH during the reaction, the pH of the pore solution decreases, making the C-S-H gel decompose [44]. The decomposition of the C-S-H gel and the formation of gypsum cause a more unfavorable influence on strength, resulting in the softening and shedding of the concrete surface. Reactions associated with magnesium attack are as follows [45]:

(5) $${\mathrm{Mg}}^{2+}+{{\mathrm{SO}}_{4}}^{2-}+{\mathrm{Ca}(\mathrm{OH})}_{2}+2H_{2}O\to {\mathrm{Mg}(\mathrm{OH})}_{2}+{\mathrm{CaSO}}_{4}\cdot 2H_{2}O$$

(6) $$3\mathrm{CaO}\cdot 2{\mathrm{SiO}}_{2}\cdot 3H_{2}O+3{\mathrm{MgSO}}_{4}+xH_{2}O\to 3{\mathrm{CaSO}}_{4}\cdot 2H_{2}O+3{\mathrm{Mg}\left(\mathrm{OH}\right)}_{2}+2{\mathrm{SiO}}_{2}\cdot xH_{2}O$$

(7) $$2{\mathrm{Mg}\left(\mathrm{OH}\right)}_{2}+2{\mathrm{SiO}}_{2}\cdot xH_{2}O\to 2\left(\mathrm{MgO}\cdot {\mathrm{SiO}}_{2}\cdot H_{2}O\right)+xH_{2}O$$

The results of TG/DTG are displayed in Figure 4. The dotted line in Figure 4 is the mass loss, and the solid line is the result of DTG. The peak at 90–120 °C is associated with the decomposition of ettringite and the dehydration of the C-S-H gel. The peak at 130–170 °C is due to the dehydration of gypsum. At 380–400 °C, the peak is generated by magnesium corrosion products, and the peak at 400–500 °C is the result of dehydration of CH. In Figure 4a, the two curves of mass loss are relatively close before 500 °C, and the peak of CH dehydration is higher at 90 days. In Figure 4b, the decline in mass loss at 90 days is more pronounced, and the generation of sulfate corrosion products also affects the content of CH. After adding BF, the pore structure is improved, thus reducing the corrosion channels for sulfate ions. Therefore, the peaks caused by the decomposition of sulfate corrosion products are decreased in Figure 4c,d. 

#### 3.2.2. Microstructures

**Figure 5:**
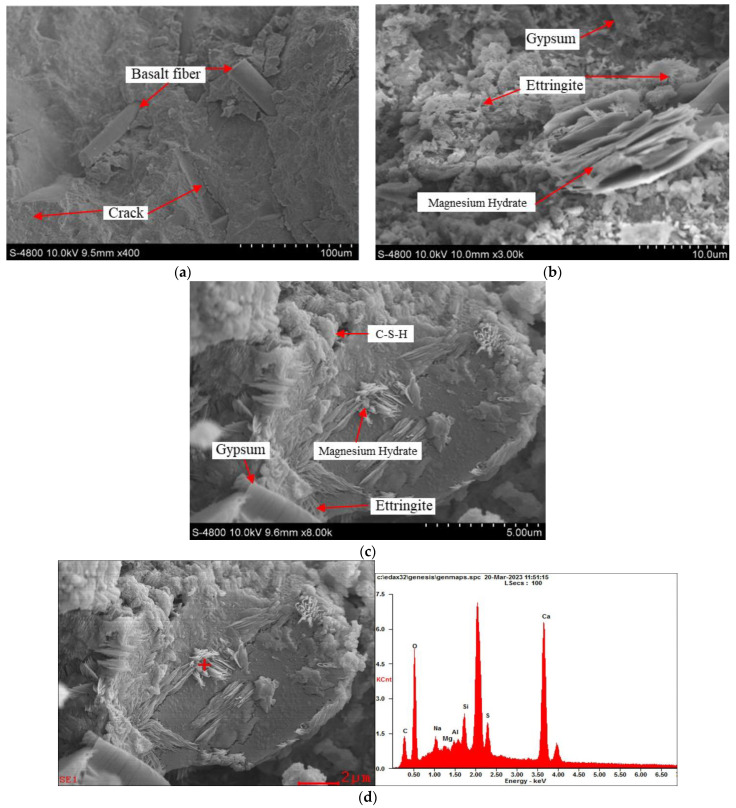

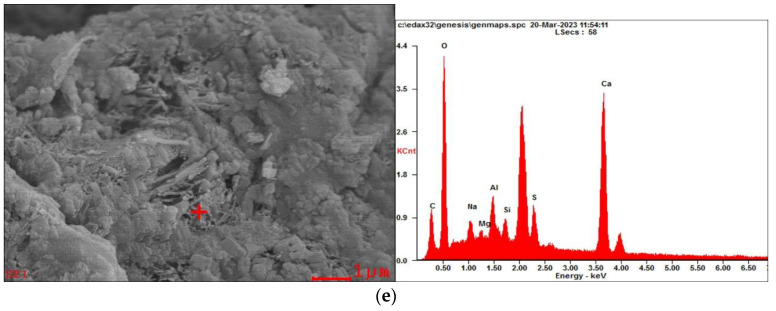
SEM and EDS results of the specimen, (**a**): S-1F, (**b**): S-M, (**c**): D-M. (**d**): S-3F, (**e**): S-5F.

Microstructural images of the specimen were obtained by SEM and the results are displayed in Figure 5. To further determine the composition of observed crystals in the SEM images, the EDS test was performed on selected areas (red cross) and results were presented together with SEM images. As depicted in Figure 5a, the original cracks provide channels for external sulfate attack. SEM images show that the shape of BF is cylindrical, and there are no clumps between the fibers, indicating excellent compatibility of BF and concrete. The surface of BF contains numerous hydroxyl groups, which would absorb water in the cement slurry to form hydrogen bonds. The existence of hydrogen bonds makes the BF embed in the cement matrix, bonding closely with the surrounding slurry, enhancing the compactness of the concrete, and filling the inherent defects. 

As displayed in Figure 5b,c, C-S-H gel in a flocculent shape would be formed during the hydration process, while ettringite in an acicular shape and gypsum in a columnar shape would be generated with the sulfate corrosion, aligning with the results of the XRD and TG analysis. Gypsum and ettringite are the main sulfate-induced corrosion products. The strength of sulfate corrosion products is relatively low and the dimension would expand, finally leading to the deterioration of the structure. Meanwhile, corrosion products induced by magnesium are mainly flaky and layered, uniformly embedding within the matrix. Figure 5d,e show the element analysis of corrosion products by EDS, the results illustrate that products contain more magnesium elements. The flaky and layered structure of magnesium hydrate blocks the channel of sulfate attack at the early stage, thereby inhibiting the early dimension change to a certain extent. When mixed with BF, the surface of BF would also accumulate hydration products. BF can enhance the strength of the matrix depending on its superior mechanical properties such as a high elastic modulus and tensile strength, coupled with well-bonding performance with concrete. On the other hand, BF has a three-dimensional random distribution system in concrete [46], which improves the crack resistance, and effectively delays the generation and expansion of internal cracks.

### 3.3. Dimension and Mass Changes

#### 3.3.1. Dimension Change

**Figure 6:**
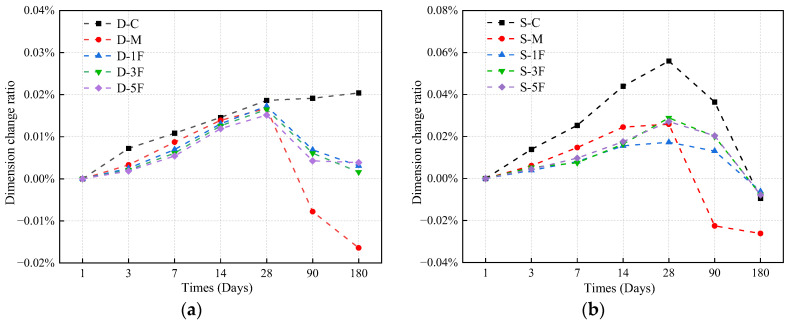
The dimension change ratio of specimens immersed in (**a**) distilled water, and (**b**) sulfate solution.

The dimension of specimens can be affected by both the generation of corrosion products and the shedding of powder and fragments from the exposed surface of specimens. The measurement of dimensions in different specimens was conducted, and the change ratio was calculated and shown in Figure 6. For specimens immersed in distilled water, the dimension of D-C increases in the early stage and tends to be stable after 28 days, while the change ratio reaches 0.020% after 180 days. The dimension change ratio of D-M is relatively low in the early stage, being 10.2% lower compared to D-C at 28 days. Corrosion induced by Mg^2+^ mainly weakens the strength rather than causes expansion [47]. With the extension of immersion time, micro-cracks and powder shedding appear on the surface due to the corrosion of premixed salts on D-M, leading to a reduction in dimension of 0.033% after 180 days. BF has a large specific surface and high elastic modulus, contributing to enhanced bond strength and the suppression of crack development in the early phase. After hardening of the specimens, there is a slight decrease in dimension for specimens premixed with BF due to powder shedding, while it tends to stabilize eventually.

For specimens immersed in sulfate solution, the dimension increase is primarily attributed to the expansive corrosion products, including gypsum and ettringite formed in the corrosion reactions. The channels formed by cracks facilitate more invasion of sulfate ions into concrete, intensifying the damage at the corners and causing more severe powder shedding on the surface, thus reducing the dimension in the later stages, just as S-C increased by 0.056% at 28 days but decreased to −0.010% after 180 days. With the influence of premixed Mg^2+^, the expansion of S-M is small in the first 28 days. However, under the long-term external sulfate–internal magnesium combined attack, the deterioration of S-M is serious and the shrinkage is great eventually. Comparing the shrinkage of specimens premixed with BF, the dimension change ratio of each specimen decreased by approximately 70% compared to S-M when immersed for 180 days. It can be considered that BF embedded in the matrix could limit the development of cracks, therefore restricting the shrinkage.

#### 3.3.2. Mass Change

**Figure 7:**
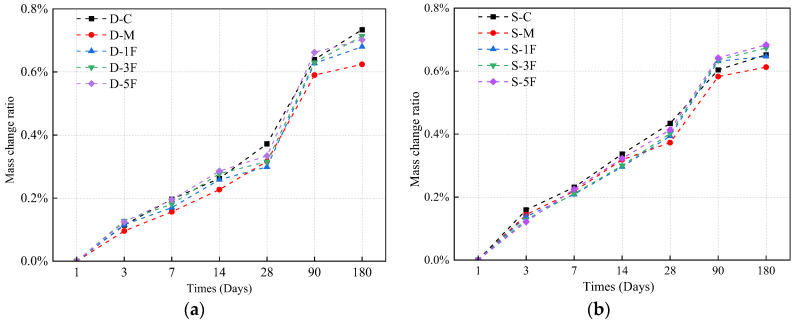
The mass change ratio of specimens immersed in (**a**) distilled water, and (**b**) sulfate solution.

The mass changing ratio of all tested specimens was calculated and the results are illustrated in Figure 7. It is evident that the degradation resulting from various corrosive conditions significantly affects the mass of the specimens.

Cast-in situ concrete is always put into the immersion solution when the hydration is far from complete. Therefore, the generation of hydration products and corrosion products leads to a substantial early-stage mass growth rate, particularly for specimens immersed in sulfate solution. The development of porosity would affect the change of mass, concrete with larger porosity (such as S-C, S-M) would bring out more pore water due to the sulfate attack, while the influence of cracks and expansion in the later stage would accelerate the falling off of concrete powder from specimens, resulting in a lower mass growth rate than that immersed in distilled water. The mass growth rate of D-M is 15% lower than D-C in the early stage, and the premixed Mg^2+^ reacts with hydration products to generate magnesium hydroxide. This reaction consumes CH, and the decrease in pH of the pore solution would cause the decomposition of the C-S-H gel. The above process continuously consumes sulfate ions, and the growth rate of corrosion products induced by sulfate would slow down when the sulfate supplementation is lacking; thus, the mass growth rate gradually reduces. The early hardening process of S-M is not completed. Under the external sulfate–internal magnesium combined attack, corrosion products embedded in the matrix would cause more weak connections within the concrete. The mass change is 5% lower compared to S-C. The incorporation of BF would increase the early densification of the concrete, thereby slightly increasing the growth rate of mass. BF would also restrain the development of crack systems and reduce the spalling in the later phase, making the mass growth rate stable.

### 3.4. Mechanical Properties

#### 3.4.1. Compressive Strength

**Figure 8:**
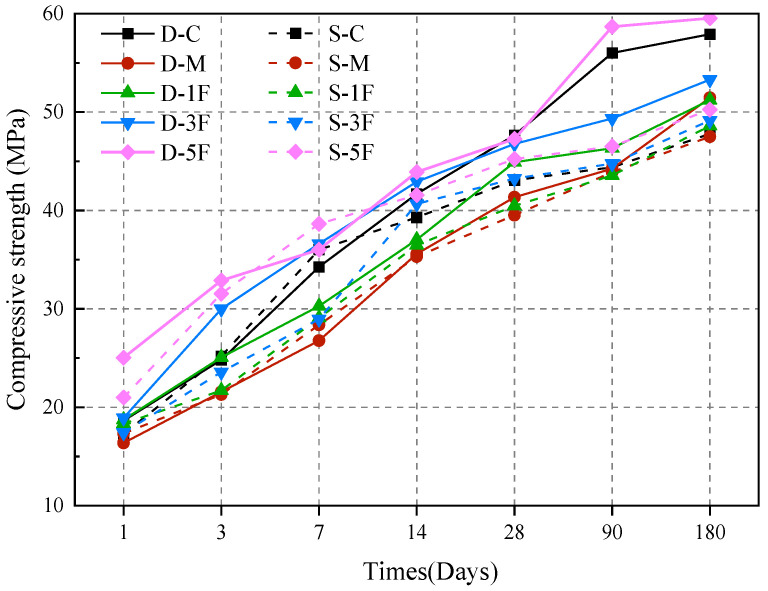
Compressive strength of specimens in different solutions.

The compressive strengths of specimens that suffered different corrosive conditions are presented in Figure 8. In general, the compressive strength of all specimens shows an increasing tendency after immersing for 180 days. The external sulfate–internal magnesium combined attack significantly reduces the compressive strength, while the added BF enhances the compressive strength.

The strength of the specimens immersed in distilled water exceeds that of those immersed in sulfate solution. For S-C, the cast-in situ concrete is attacked by external sulfate when the hydration is not completed. During the initial stages of corrosion, the corrosion products fill the internal pores, increasing the compactness of the specimen, and making the compressive strength slightly higher than D-C; the growth rate is higher in the initial 7 days as well. However, with the increase in immersion time, corrosion products accumulate within cracks and pores, which promotes the extension of these cracks. The compressive strength of S-C enhances mildly after 28 days, and the strength at 180 days only accounts for 82.54% of D-C. For specimens premixed with MgSO_4_, the deterioration caused by corrosion is more serious. As illustrated in Figure 8, the compressive strength of D-M and S-M is relatively low during the early stage, and the strength at 28 days is decreased by 13.2% and 17.2% compared to D-C, respectively. On account of S-M being attacked by external sulfate, the filling of corrosion products makes the compressive strength higher than D-M in the early days. However, with the prolonged immersion time, the expansion stress caused by the corrosion products makes the internal cracks develop continuously, so the compressive strength of S-M is low after 28 days. Previous studies have shown that corrosion products induced by Mg^2+^ are embedded in the matrix rather than accumulated in the pores, thus blocking the cracks and preventing the rapid diffusion of sulfate, lowering the compactness of the specimen at the early stage. CH generated during the hydration process can react with Mg^2+^ and SO_4_^2−^, decreasing the pH of the pore solution and making the C-S-H gel unstable [48]. There is a competitive relationship between premixed Mg^2+^ and SO_4_^2−^. Due to Mg^2+^ and Ca^2+^ having the same valence state and similar ionic radius, it is easier for Mg^2+^ to have a limited magnesium–calcium exchange with C-S-H gel to generate M-S-H with lower strength [49,50]. In addition, during the initial phase of cement hydration, premixed Mg^2+^ can decrease the specific surface area of cement, therefore hindering the hydration process, generating Tless AFt and CH 20]. Therefore, it can be considered that both internal and external corrosive ions have obvious negative effects on the development of compressive strength.

There are plenty of original pores in cast-in situ concrete. BF has a small diameter and excellent bonding effect, and can fill the pores and improve the pore structure. After 3 days, the compressive strength of D-1F, D-3F, and D-5F is 16.28%, 39.19%, and 52.50% higher than D-M, respectively. After 180 days, the strength of D-1F is close to D-M, while D-5F is even higher than D-C. For specimens exposed to external sulfate attack, the filling of pores by BF reduces the accumulation of corrosion products and weakens the erosion effect on the matrix to an extent. On the other hand, BF can transfer the stress and bridge the matrix. At 180 days, the strength of S-1F, S-3F, and S-5F exhibits an increase of 20.08%, 13.64%, and 11.09%, respectively, compared with 28 days. Overall, the compressive strength of cast-in situ concrete is enhanced with the increment of BF content under the external sulfate–internal magnesium combined attack. 

#### 3.4.2. Flexural Strength

**Figure 9:**
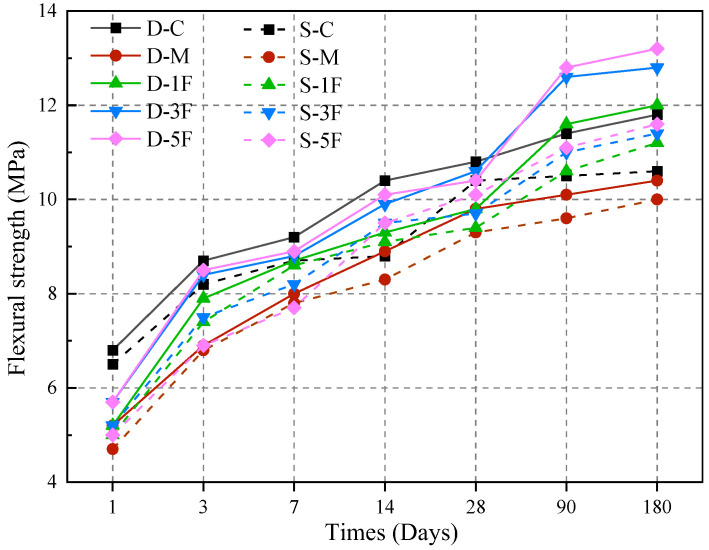
Flexural strength of specimens in different solutions.

The flexural strength of all specimens was determined by a three-point bending test, and the results are given in Figure 9. The results obviously indicate that the flexural strength of cast-in situ concrete is heavily affected by corrosive environments and premixed BF.

As shown in Figure 9, the flexural strength of all specimens increases at 180 days, and the strength of concrete immersed in sulfate solution is lower than that immersed in distilled water. In the initial corrosion stage, the hydration reaction and corrosion reaction occur simultaneously. As the hydration products are generated, the specimen becomes more compact, and the growth rate of flexural strength is relatively high. However, corrosion products induced by both magnesium and sulfate would also be generated, and the flexural strength of D-M at 28 days is 9.3% lower than that of D-C, indicating the premixed corrosive MgSO_4_ would decrease the strength in the early stage. External corrosion caused by sulfate results in a lower strength for S-M, which decreases by 14.1% compared to D-C. Under long-time immersion, the strength of D-M and S-M significantly decreased by 11.8% and 15.3%, respectively, compared with D-C; the growth rate is slower as well. 

After immersing for 90 days, the flexural strength of specimens premixed with BF is significantly superior to that of D-M and S-M. BF has high tensile strength and a large elastic modulus. In corrosive environments, cracks usually spread along the weak points inside the concrete. When BF is evenly dispersed into the concrete, it prevents the propagation of micro-cracks during the hardening stage. The bridging effect of the fibers can transfer the received force to both sides of the fiber, reducing the stress concentration at the crack tip inside the concrete, therefore improving the flexural strength. On the whole, S-5F exhibits the best strengthening effect, with a 16.2% higher flexural strength compared to S-M at 180 days. Due to the increased density and the reinforcement provided by BF, the strength of D-1F, D-3F, and D-5F at 90 days increases by 18.36%, 18.86%, and 23.07%, respectively, compared with 28 days, while the growth rate decreases sharply after 90 days. In comparison, both the flexural strength and growth rate of concrete submerged in sulfate solution are lower. It can be speculated that the corrosion products induced by sulfate exert continuous pressure on the inner wall of the concrete, which causes an unfavorable influence on flexural strength.

### 3.5. Sulfate Concentration

**Figure 10:**
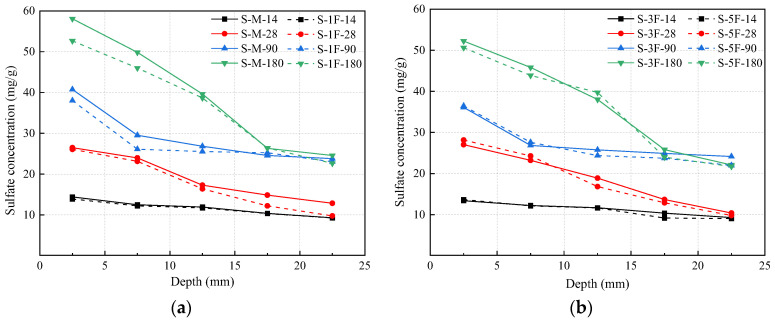
Sulfate concentrations of specimens at different immersion times: (**a**) S-M and S-1F, and (**b**) S-3F and S-5F. The numerals represent the immersion times.

Sulfate concentrations at different depths for different specimens were measured using chemical titration and the results are presented in Figure 10. The powder was drilled and collected every 5 mm, and the abscissa in Figure 10 is the average value of the drilling. The hydration of cast-in situ concrete is incomplete during the early stage, and the presence of original defects and pores causes the rapid diffusion of sulfate. As shown in Figure 10, a declining tendency of sulfate concentration can be observed with the increase in depth beneath the exposed surface of specimens. The concentration at 0–5 mm increases significantly at 180 days, while the concentration at 20–25 mm is similar to the data at 90 days. Generally, the appearance change would affect the sulfate diffusion. A crack in the concrete multiplies the diffusion path for corrosive ions and accelerates the degradation. Specimens premixed with Mg^2+^ have a more serious degree of degradation, as shown in Figure 2, which correspondingly accelerates the sulfate diffusion rate, leading to a high sulfate concentration as a result. As mentioned above, the incorporation of BF limits the progression of the crack system and mitigates the corrosion channels of external sulfate to a certain extent. Therefore, the sulfate concentration of the concrete premixed with BF is lower than S-M.

## 4. Discussion

The physical and mechanical properties of concrete are constitutionally determined and affected by mineral and microstructural changes. For specimens immersed in sulfate solution, the accumulation of corrosion products induced by sulfate can lead to the falling off and expansion of the specimen. As microcracks multiply, more corrosive ions from the external environment infiltrate the specimen, causing an increase in internal stress and resulting in severe deterioration. When the internal stress exceeds the tensile strength, the specimen will undergo destruction and failure [51,52]. Combining changes in the physical and mechanical properties of the D-M and S-M specimens, the premixed magnesium salt primarily affects the strength rather than causing expansion in the early stage. Layered magnesium corrosion products were embedded in the matrix and cracks, blocking the channels for external sulfate ions and weakening the filling of pores by gypsum and ettringite. As a result, there are minimal changes in dimension and mass, while the formation of non-cementing M-S-H gel also affects the development of strength. As sulfate ions continued to invade, a large amount of corrosion products was generated within the specimen, resulting in a significant decrease in the mechanical properties in the later stage of corrosion.

The physical restraint provided by admixture in concrete would impact the strength development and degradation process when exposed to an aggressive environment. As a widely used inorganic fiber, BF has high tensile strength and excellent toughness, and adding an appropriate content of BF would positively affect the properties of the concrete. BF does not react with the corrosive ions, and the corrosion products attached to the slurry and fiber do not destroy the fiber structure as well. After being premixed, BF bonds closely with the concrete, fills the holes, increases the compactness, and reduces the channels of external sulfate, therefore improving the compressive strength and flexural strength as evident in Figure 8 and Figure 9. Under the external sulfate–internal magnesium combined attack, BF mainly exerts the effect of strengthen and crack resistance. For the strengthening effect, the fiber improves the microstructure of the matrix and reduces the internal defects. The proper amount of fiber bonds with the matrix effectively, and the bridging effect enhances the load-bearing capacity. The mechanical properties of the concrete are most obviously improved by 0.5% fiber under the combined attack. For the crack-resistance effect, the proper amount of BF prevents the formation and expansion of microcracks during the hardening process, and can also reduce the stress concentration in the crack tip of the concrete. BF has a three-dimensional random distribution system in concrete due to its high elastic modulus, therefore improving the overall cracking resistance.

## 5. Conclusions

Experiments were conducted to reveal the effects of BF on the durability behavior of cast-in situ concrete exposed to internal magnesium and external sulfate attack. Physical, chemical, and mechanical properties were continuously determined during the immersion time. Mineral and microstructural changes were observed by XRD, TG, and SEM analysis methods to illustrate the degradation mechanism. The following observations can be made:

1. Corrosion products induced by magnesium primarily affect the strength instead of causing the expansion. Under the combined attack of external sulfate–internal magnesium, the specimen exhibits more severe degradation and relatively poor mechanical performance.
2. After premixing with BF, the physical and mechanical properties of cast-in situ concrete are improved. BF mainly exerts the effects of strengthening and improving crack resistance. For strengthening, BF fills in the original defects and enhances the load-bearing capacity of the specimens. In terms of crack resistance, it restricts the development of cracks and reduces stress concentration at the crack tip.
3. A 0.5% content of BF results in the most significant improvement in the properties of specimens. Under the attack of internal magnesium salt, both the compressive and flexural strength of these samples are higher than the control samples at 180 days. When exposed to external sulfate–internal magnesium combined attack for a long period, the flexural strength of specimens with 0.5% BF increased by 16.2%.
4. Moreover, there is no available model for the development of strength under the external sulfate–internal magnesium combined attack. In the follow-up research, we will focus on carrying out statistical tests and parameter analysis to build applicable models. We will continue to research the performance of basalt fiber-reinforced concrete with different lengths and contents of BF, aiming to provide more references for the application of BF in concrete.

## Figures and Tables

**Table 1:** Cement composition and basalt fiber physical properties.

Components of Cement	CaO	SiO_2_	Al_2_O_3_	Fe_2_O_3_	SO_3_	MgO	K_2_O	TiO _2_	Na_2_O	Cl
Content (%)	67.21	18.48	5.64	3.63	2.40	0.75	0.68	0.51	0.31	0.08
Physical properties of basalt fiber	Length		Diameter		Tensile strength		Elastic modulus		Density	
Index	12 mm		17 µm		3000–4800 MPa		90–100 GPa		2.80 gꞏcm^−3^	

**Table 2:** Detailed information on the specimens.

Specimens in Distilled Water	Specimens in Sulfate Solutions	Premixed Salts	Volume of BF
D-C	S-C	No premixed salts	0
D-M	S-M	3% MgSO_4_	0
D-1F	S-1F	3% MgSO_4_	0.1%
D-3F	S-3F	3% MgSO_4_	0.3%
D-5F	S-5F	3% MgSO_4_	0.5%

## Data Availability

The raw/processed data required to reproduce these findings cannot be shared at this time as the data also form part of an ongoing study.
